# Three-layer microfibrous peripheral nerve guide conduit composed of elastin-laminin mimetic artificial protein and poly(L-lactic acid)

**DOI:** 10.3389/fchem.2014.00052

**Published:** 2014-07-18

**Authors:** Sachiro Kakinoki, Midori Nakayama, Toshiyuki Moritan, Tetsuji Yamaoka

**Affiliations:** ^1^Department of Biomedical Engineering, National Cerebral and Cardiovascular Center Research InstituteSuita, Japan; ^2^Department of Clinical Engineering, Faculty of Medical Engineering, Suzuka University of Medical ScienceSuzuka, Japan

**Keywords:** poly(L-lactic acid), elastin-laminin mimetic protein, electrospun microfiber, nerve conduit, tissue adhesion prevention

## Abstract

We developed a microfibrous poly(L-lactic acid) (PLLA) nerve conduit with a three-layered structure to simultaneously enhance nerve regeneration and prevent adhesion of surrounding tissue. The inner layer was composed of PLLA microfiber containing 25% elastin-laminin mimetic protein (AG73-(VPGIG)_30_) that promotes neurite outgrowth. The thickest middle layer was constructed of pure PLLA microfibers that impart the large mechanical strength to the conduit. A 10% poly(ethylene glycol) was added to the outer layer to prevent the adhesion with the surrounding tissue. The AG73-(VPGIG)_30_ compositing of an elastin-like repetitive sequence (VPGIG)_30_ and a laminin-derived sequence (RKRLQVQLSIRT: AG73) was biosynthesized using *Escherichia coli*. The PLLA microfibrous conduits were fabricated using an electrospinning procedure. AG73-(VPGIG)_30_ was successfully mixed in the PLLA microfibers, and the PLLA/AG73-(VPGIG)_30_ microfibers were stable under physiological conditions. The PLLA/AG73-(VPGIG)_30_ microfibers enhanced adhesion and neurite outgrowth of PC12 cells. The electrospun microfibrous conduit with a three-layered structure was implanted for bridging a 2.0-cm gap in the tibial nerve of a rabbit. Two months after implantation, no adhesion of surrounding tissue was observed, and the action potential was slightly improved in the nerve conduit with the PLLA/AG73-(VPGIG)_30_ inner layer.

## Introduction

Traumatic nerve injuries in which direct suturing of the proximal and distal stumps is difficult due to the long nerve gap are generally treated with nerve autografts. However, autologous nerve grafting has several disadvantages, such as size mismatch and permanent loss of donor function due to the extraction of normal nerve. As an alternative, artificial nerve conduits have become widely accepted for bridging gaps between nerve stumps (Ichihara et al., 2008; Lohmeyer et al., 2009; Sieminow and Brzezicki, 2009).

Artificial nerve conduits constructed of numerous polymeric materials such as silicone (Lundborg et al., 1982), collagen (Archibald et al., 1995), chitosan (Freier et al., 2005; Ao et al., 2006), hyaluronic acid (Wang et al., 1998), poly(caprolactone) (PCL), poly(glycolic acid) (PGA), and poly(lactic acid) (PLA) (Nakamura et al., 2004; Yoshitani et al., 2007) have been investigated. Recently, an artificial nerve conduit composed of poly(lactic-*co*-glycolic acid) (PLGA) mesh filled with animal-derived collagen has been put into clinical use and has shown good performance (Nakamura et al., 2004). PLGA is hydrolyzed and metabolized *in vivo*, and thus the PLGA conduit is absorbed during nerve regeneration over the course of several months (Mainil-Varlet et al., 1996). Because PLGA does not possess any biological activity, collagen is used for promoting nerve regeneration. Collagen is a major component of the basement membrane of nerve tissue and plays a key role in the reconstruction of the axon network by Schwann cells (Thomas, 1964; Chernousov et al., 2008). Although collagen strongly enhances nerve regeneration, animal-derived materials raise concerns about viral infection and immune responses (Lynn et al., 2004). The mesh structure of this conduit is effective for inhibiting the intrusion of surrounding connective tissues and for allowing the permeation of liquid factors and small molecules. However, the mesh structure promotes adherence with surrounding tissues and can cause painful traction neuropathies (Smit et al., 2004). Hence, the ideal conduits for promoting nerve regeneration and preventing tissue adhesion would not include animal-derived materials.

To avoid the use of animal-derived materials, short peptide sequences isolated from extracellular matrix proteins can be used, because they show biological activity equal to that of full-length proteins. For example, the RGD sequence of fibronectin provides excellent cell adhesive properties (Hersel et al., 2003). Focusing on nerve regeneration, the IKVAV (Tashiro et al., 1989), YIGSR (Iwamoto et al., 1987), and RKRLQVQLSIRT (AG73) (Nomizu et al., 1996) sequences derived from laminin have been well studied in the activation of neural cells. These small peptides are easily synthesized chemically or biologically and are useful for imparting nerve regenerative activity to polymeric materials (Rao and Winter, 2009; Kakinoki and Yamaoka, 2010). Suzuki et al. prepared IKVAV- or YIGSR-immobilized tendon chitosan tubes and demonstrated the efficacy of peptide immobilization for assisting nerve regeneration in a rat model of nerve injury (Suzuki et al., 2003a). Previously, we reported that poly(l-lactic acid) (PLLA) nerve conduits modified with laminin-derived AG73 peptides and a polyethylene glycol (PEG)-containing outer layer are effective for preventing the adhesion of surrounding tissue (Kakinoki et al., 2011). The AG73 peptide was immobilized onto PLLA microfibers using oligo(d-lactic acid) (ODLA)–AG73 conjugates. The microfibers were fabricated by electrospinning of a mixed solution containing PLLA and the ODLA–AG73 conjugate, resulting in stable immobilization of the AG73 peptide via stereocomplex formation between the PLLA and the ODLA. Regeneration of functional nerve tissue was observed in AG73-immobilized microfibrous nerve conduit in the rat model of nerve injury, but it required a long period of time, approximately 6 months. Thus, the efficiency of AG73-immobilized microfibers is insufficient for nerve regeneration. We speculated that the efficacy of AG73 immobilization was decreased immediately after implantation by release of the low-molecular weight PDLA–AG73 conjugates from the microfibers *in vivo*.

To fabricate a more effective biological material for nerve regeneration, we designed and biosynthesized a high molecular weight elastin-laminin mimetic protein. The basement membrane of nerve tissue is primarily constructed with collagen, laminin, and elastin (Rutka et al., 1988). These proteins cooperatively support the process of nerve regeneration. Specifically, collagen and laminin function in axon extension, and elastin provides elasticity for the tissue structure (Toyota et al., 1990). We previously biosynthesized an elastin-laminin mimetic protein and used it for the functionalization of PLLA scaffolds (Kakinoki and Yamaoka, 2014). This protein is composed of 30 repeats of an elastin-like VPGIG repetitive sequence and a laminin-derived AG73 sequence (AG73-(VPGIG)_30_). AG73-(VPGIG)_30_ showed temperature-dependent coacervation in phosphate buffered saline (PBS) solution at approximately 14°C. Because the AG73-(VPGIG)_30_ was insoluble at body temperature, this protein was expected to serve as a suitable scaffold for nerve regeneration. Our results showed that the adhesion and neurite outgrowth of PC12 cells were enhanced on the AG73-(VPGIG)_30_ adsorbed PLLA films *in vitro*.

In this study, we prepared PLLA microfibrous nerve conduit with a three-layered structure that promoted nerve regeneration and prevented tissue adhesion. The therapeutic efficacy of peripheral nerve regeneration was evaluated using a rabbit model of nerve injury. Microfibrous conduits composed of a PLLA, PLLA/AG73, or PLLA/AG73-(VPGIG)_30_ inner layer, a PLLA middle layer, and a PLLA/PEG outer layer were fabricated using an electrospinning procedure. Adhesion and neurite outgrowth of PC12 cells on PLLA microfibers containing AG73-(VPGIG)_30_ were studied *in vitro*. Nerve autografts and microfibrous conduits were implanted in rabbits to bridge a 2-cm tibial nerve gap. Two months after implantation, nerve regeneration was evaluated by electrophysiological measurements.

## Materials and methods

### Expression and purification of the elastin-laminin mimetic protein

The elastin-laminin mimetic protein (AG73-(VPGIG)_30_) was expressed by *Escherichia coli* BL21(DE3)pLysS (Life Technologies Corporation, Carlsbad, CA, USA) that had been transformed with the pET28(+) vector (Merck KGaA, Darmstadt, Germany) encoding AG73-(VPGIG)_30_, as described previously (Kakinoki and Yamaoka, 2014). AG73-(VPGIG)_30_ expression was automatically induced using an Overnight Express™ Autoinduction System (Merck KGaA). Briefly, *E. coli* cells were incubated in 2 × YT medium supplemented with 34 μg/mL of kanamycin and the reagents of the Overnight Express™ Autoinduction System at 30°C for 24 h. *E. coli* was harvested by centrifugation at 3500 × *g* at 4°C for 15 min. Bacterial pellets were resuspended in lysis solution (8 M urea) and frozen at −80°C. After thawing, bacteria were disrupted by sonication on ice. Insoluble debris was removed by centrifugation at 10,000 × *g* at 4°C for 15 min, and the supernatant was purified on a His-tag affinity column (COSMOGEL His-Accept, Nacalai Tesque, Kyoto, Japan). Following dialysis (MwCo = 10,000 Da) in deionized water at 4°C, purified AG73-(VPGIG)_30_ was obtained by lyophilization. The purity of the AG73-(VPGIG)_30_ was confirmed by sodium dodecyl sulfate-polyacrylamide gel electrophoresis (SDS-PAGE) analysis with silver staining.

### Fabrication of PLLA/AG73-(VPGIG)_30_ non-woven microfibers

A PLLA/AG73-(VPGIG)_30_ solution with a concentration of 20w% was prepared by dissolving PLLA (Mw: 106,000, Mn: 60,000, Mw/Mn: 1.77) (Musashino Chemical Laboratory, Inc., Tokyo, Japan) and AG73-(VPGIG)_30_ in hexafluoroisopropanol (HFIP) at a weight ratio of 4:1. This solution was electrospun using a plastic syringe equipped with a stainless steel needle (length = 15.0 mm, diameter = 20 G) at a constant feed rate of 4 mL/h. An aluminum plate was used as a target and the distance between the target and the needle tip was 100 mm. The solution was electrospun at a high voltage (15 kV) for 5 min. Non-woven PLLA/AG73-(VPGIG)_30_ microfibers were collected and cut for subsequent experiments. Non-woven PLLA and PLLA/AG73 microfibers were prepared using a similar procedure. The concentration of the AG73 peptide (Purity > 82.6%; Sigma-Aldrich, Inc.) in the PLLA/AG73 solution was adjusted to the same molarity as that of the AG73-(VPGIG)_30_ in the non-woven PLLA/AG73-(VPGIG)_30_ microfibers. After immersion in PBS for 24 h at 37°C, the structure of the non-woven microfibers was observed by scanning electron microscopy (SEM; JCM-5700, JEOL, Tokyo, Japan).

### Neurite outgrowth assay

A neurite outgrowth assay was performed using rat adrenal pheochromocytoma (PC12) cells (RIKEN BioResorce Center, Ibaraki, Japan), which are widely used as a model for neural stem cells. PC12 cells were maintained in Dulbecco's modified Eagles medium (DMEM) supplemented with 100 units/mL penicillin, 100 μg/mL streptomycin (Life Technologies Corporation, Carlsbad, CA, USA), 10% fetal bovine serum (FBS; MP Biomedicals, Inc., Solon, OH, USA), and 7.5% horse serum (HS; Sigma-Aldrich, Inc., St. Louis, MO, USA). PC12 cells were cultured in poly-d-lysine-coated cell culture dishes (Asahi Glass Co., Ltd., Tokyo, Japan) and maintained at 37°C in a 5% CO_2_ atmosphere. Prior to the neurite growth assay, PC12 cells were cultivated in DMEM/F12 medium (Life Technologies Corporation, Carlsbad, CA, USA) with 100 ng/mL nerve growth factor (NGF; Sigma-Aldrich, Inc.) for 24 h on polystyrene cell culture dishes. The medium was refreshed with normal culture medium, and cells were incubated for 30 min at 37°C in a 5% CO_2_ atmosphere. Cells were harvested by gentle agitation and resuspended in advanced DMEM/F12 containing 5 mg/mL insulin (Life Technologies Corporation, Carlsbad, CA, USA), 100 ng/mL NGF, 20 nM progesterone, 100 mg/mL transferrin, and 30 nM sodium selenite (Na_2_SeO_3_) (Nacalai Tesque, Inc., Kyoto, Japan), and seeded on non-woven microfibers fixed with Cell Crown (Scaffdex Ltd., Tampere, Finland) in 24-well cell culture plates at a density of 2.0 × 10^4^ cells/sample. After 48 h incubation at 37°C in a 5% CO_2_ atmosphere, cells adherent to the non-woven fibers were fixed with 10% formalin and stained with 4% crystal violet/methanol solution. The number of adherent cells observed in the stained images were counted and categorized according to neurite length.

### Fabrication of PLLA/AG73-(VPGIG)_30_ microfibrous conduit

We designed the electrospun microfibrous conduits with 3 layer structure, PLLA, PLLA/AG73, or PLLA/AG73-(VPGIG)_30_ inner layer, PLLA middle layer, and PLLA/polyethylene glycol (PEG) outer layer. For fabricating conduits, a rotating stainless steel tube (outer diameter = 2.0 mm, speed = 1500 rpm) was used as a target. Other conditions for electrospinning were completely same to nonwovens. First, inner layer (PLLA, PLLA/AG73, or PLLA/AG73-(VPGIG)_30_) was electrospun as described previously for 5 min. Middle layer was electrospun with 20w% of PLLA solution for 30 min. Then, the mixed solution at 20w% containing PLLA and PEG (9:1) was electrospun for 10 min as an outer layer. The matrices mass for inner, middle, and outer layer were adjusted to 1: 6: 2 for covering inner and outer surface of the mechanically strong middle layer. Microfibrous conduits were cut to length of 22 mm, and immersed in 70%-ethanol for the sterilization. After drying *in vacuo*, conduits were used for animal experiment.

### Implantation of microfibrous conduits

Electrospun microfibrous conduits were implanted into 2.0-cm gaps in the left tibial nerve of a total of 12 New Zealand white rabbits (3.0–3.5 kg, male) (Oriental Yeast Co., Ltd., Tokyo, Japan). This study was performed in accordance with the animal experimental guidelines of the National Cerebral and Cardiovascular Center Research Institute. Rabbits were anesthetized by intramuscular injection of 0.1 mL/kg of Selactar (Bayer Yakuhin, Ltd., Osaka, Japan) and anesthesia was maintained by inhalation of 3% Escain isoflurane (Mylan Inc., Canonsburg, PA, USA). Under an operating microscope, the left tibial nerve was exposed and a 2.0-cm segment was removed. A section of microfibrous conduit was filled with physiological saline solution and sutured with 10-0 vicryl (Ethicon, Somerville, NJ, USA) to bridge the gap between the proximal and distal stumps. Both nerve stumps were pulled 1.0 mm inside the conduits. In addition, the removed nerve tissue was inverted on its proximal–distal axis and implanted as an autograft control. Muscle and skin were closed with 3-0 silk sutures (Ethicon), and the rabbits were allowed to recover in a controlled environment.

### Electrophysiological analysis

Two months after implantation, nerve regeneration was evaluated by electrophysiological analysis. Rabbits were anesthetized in the same manner as for transplantation of the microfibrous conduits, and the implanted site was exposed from the proximal to the distal portions. In order to record electromyograms at the implantation site, a pair of stimulating and recording electrodes was attached to the proximal and distal portions, respectively. Both electrodes were connected to an electric stimulator (SEN-3401, Nihon Kohden, Tokyo, Japan) and a data acquisition system (PowerLab 8/30, ADInstruments, Colorado Springs, CO, USA). The stimulation parameters were as follows: strength = 1 V, current = 1 mA, duration = 10 s, pulse width = 0.1 ms. The active potential was amplified using a PowerLab system (ADInstruments, Burlingame, CA, USA), and recorded as the average of 50 traces.

## Results and discussion

### Expression and purification of elastin-laminin mimetic protein

**Figure 1:**
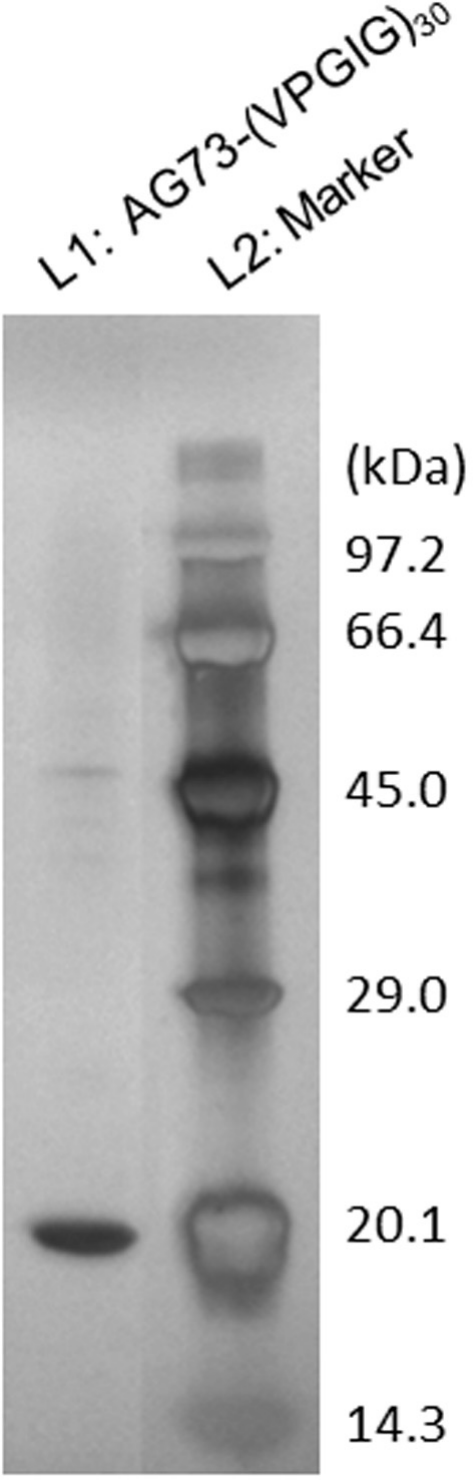
**Silver staining of the gel after SDS-PAGE of purified AG73-(VPGIG)_30_**.

SDS-PAGE analysis of purified AG73-(VPGIG)_30_ is shown in Figure 1. A single band was observed at 17–18 kDa, which is concordant with the theoretical molecular weight. Sixty milligrams of high-purity AG73-(VPGIG)_30_ was successfully obtained from 1 L of culture medium. As previously reported, PBS solution containing AG73-(VPGIG)_30_ demonstrated temperature-dependent coacervation at 14°C, indicating that the protein is insoluble at 37°C in a physiological environment (Kakinoki and Yamaoka, 2014).

### Morphology and stability of electrospun non-woven microfibers

**Figure 2:**
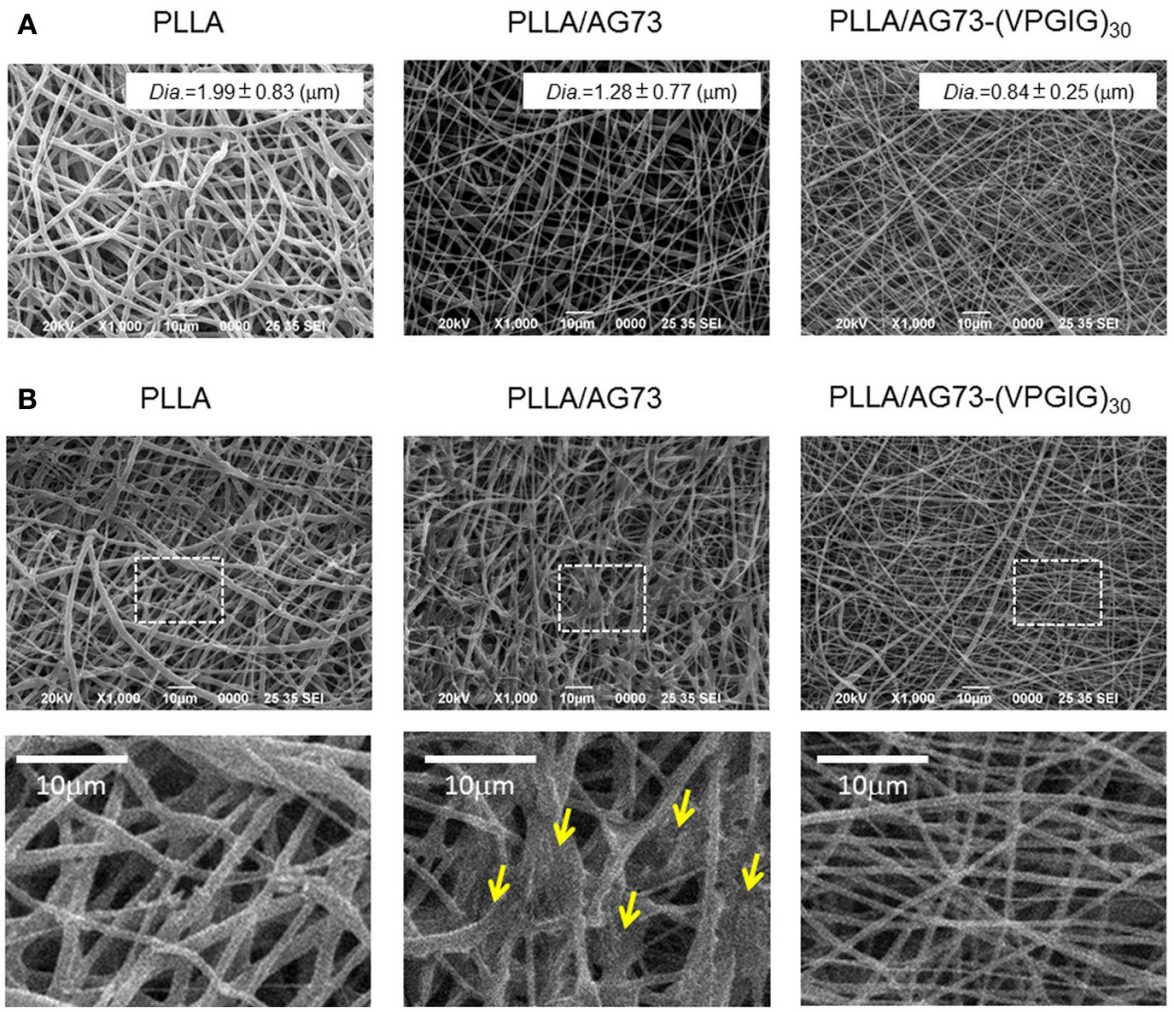
**SEM images of electrospun PLLA, PLLA/AG73, and PLLA/ AG73-(VPGIG)_30_ micro-fibers (A) before and (B) after immersion in PBS for 24 h at 37°C. Yellow arrows indicate fusion points**.

SEM images of PLLA, PLLA/AG73, and PLLA/AG73-(VPGIG)_30_ non-woven microfibers before and after PBS immersion are shown in Figure 2. The diameter of the PLLA microfibers was approximately 2.0 μm (Figure 2A). When AG73 or AG73-(VPGIG)_30_ was mixed with the PLLA, the diameter of the microfibers decreased to approximately 1.3 and 0.9 μm, respectively. The diameter of electrospun microfibers is known to be influenced by solution viscosity and electronic charge (Huang et al., 2003). The PLLA concentration was decreased by the addition of the AG73 and AG73-(VPGIG)_30_, thus decreasing the viscosity of the solution. Furthermore, the electronic charge of the solution should be positive because AG73 is positive due to its Arg and Lys residues. We assumed that the diameter of the microfibers was decreased by the changes in solution viscosity and electronic charge with the addition of the AG73 and AG73-(VPGIG)_30_. The surfaces of the PLLA/AG73 and PLLA/AG73-(VPGIG)_30_ were smooth, without phase separation, suggesting that the AG73 and AG73-(VPGIG)_30_ were homogeneously mixed.

The morphology of the microfibers following immersion in PBS for 24 h is shown in Figure 2B. The shape of the PLLA microfibers did not differ before and after immersion in PBS. However, the morphology of PLLA/AG73 microfibers became rough and some fusion appeared (Figure 2B; indicated with yellow arrows) after PBS immersion. These morphological changes suggested that the PLLA/AG73 microfibers partially dissolved in PBS. In contrast, the morphology of PLLA/AG73-(VPGIG)_30_ microfibers did not change after PBS immersion, indicating that the PLLA/AG73-(VPGIG)_30_ microfibers were stable in a physiological environment.

### Neurite outgrowth of Pc12 cells on non-woven microfibers

**Figure 3:**
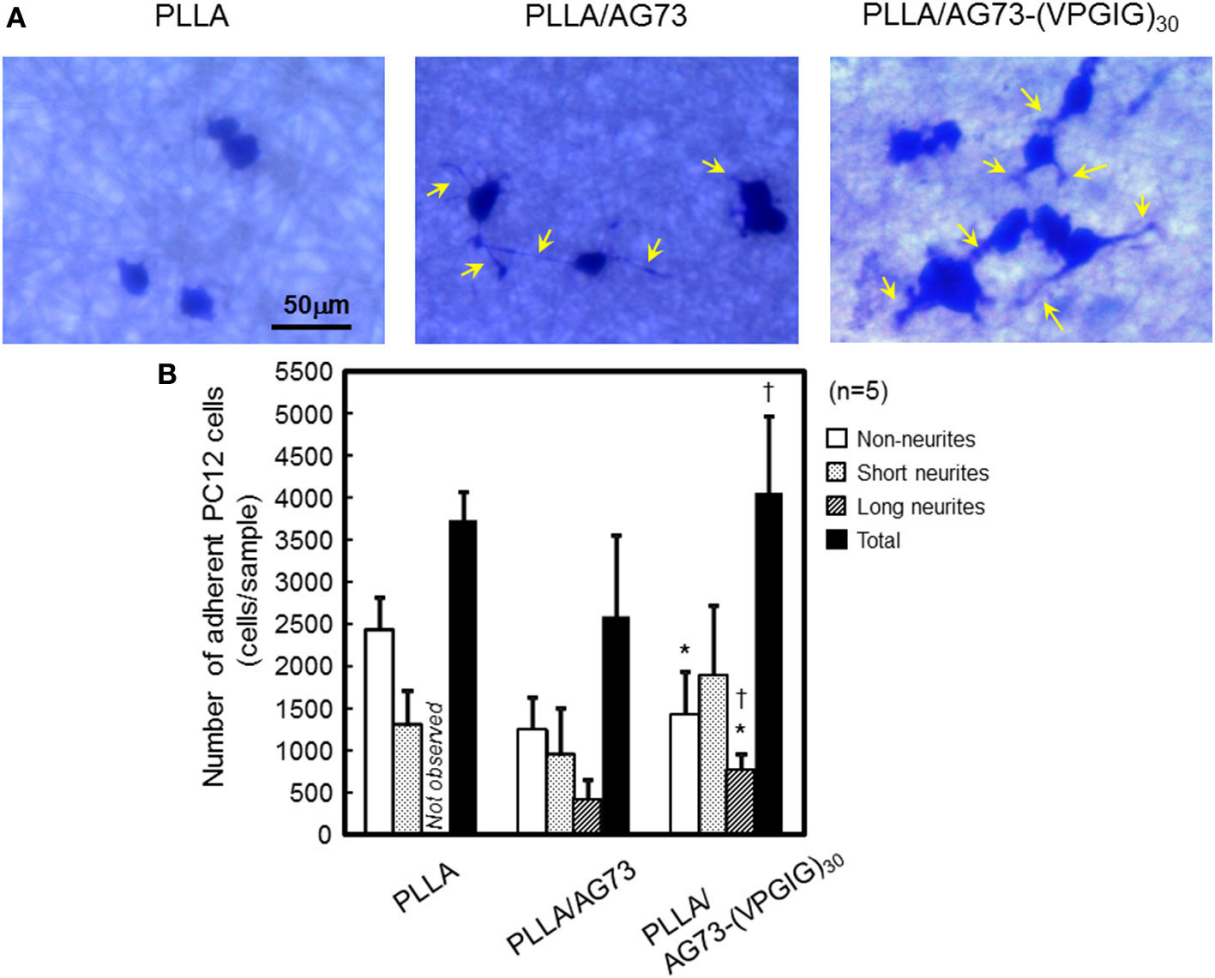
**Adhesion and neurite outgrowth of NGF-treated PC12 cells on electrospun nanofibers**. **(A)** Morphology of NGF-treated PC12 cells 48 h after seeding. Yellow arrows indicate neurites. **(B)** The number of adherent PC12 cells categorized as non-neurites, short neurites (< 50 μm), and long neurites (≥ 50 μm). ^*^ and ^†^ indicate statistically significant differences, *p* < 0.05 vs. PLLA and PLLA/AG73, respectively (Student's *t*-test).

The morphology and number of PC12 cells adherent on non-woven microfibers are shown in Figure 3. On PLLA, the rate of adherence of PC12 cells was approximately 3750/sample. Most of the adherent cells were non-neurites, and cells with long neurites were not observed. On PLLA/AG73, a similar number of adherent cells was observed, but neurite outgrowth was slightly enhanced in comparison to PLLA. PC12 cells have been reported to adhere to AG73-immobilized substrates through syndecans and the NGF pathway has been reported to be activated, resulting in neurite outgrowth (Suzuki et al., 2003b). Thus, AG73 must be stably immobilized on a substrate to express its biological function. The AG73 was removed from the surface of the PLLA/AG73 microfibers in culture medium, resulting in poor enhancement of adhesion and neurite outgrowth of PC12 cells. On PLLA/AG73-(VPGIG)_30_ microfibers, the adhesion and neurite outgrowth of PC12 cells was significantly accelerated compared to PLLA and PLLA/AG73. Because the hydrophobicity of the (VPGIG)_30_ region renders AG73-(VPGIG)_30_ insoluble in PBS, it was stably bound to the PLLA. The hydrophilic AG73 region was available to contact PC12 cells at the outermost surface of the microfibers, resulting in promotion of adhesion and neurite outgrowth of PC12 cells.

### Nerve regeneration in the rabbit model of nerve injury

**Figure 4:**
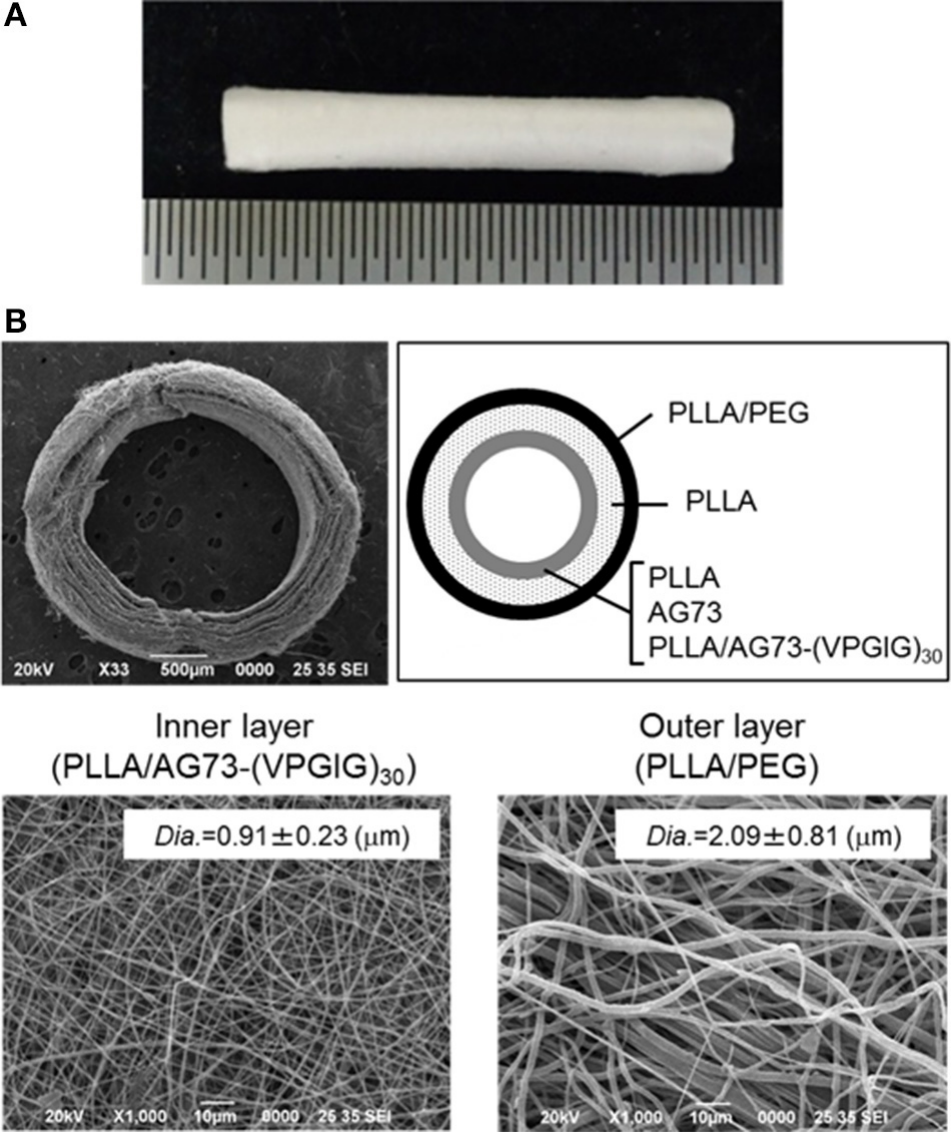
**Micro-fibrous nerve conduit with a three-layered structure**. **(A)** Whole image, **(B)** SEM images of cross-sections and inner and outer layers.

**Figure 5:**
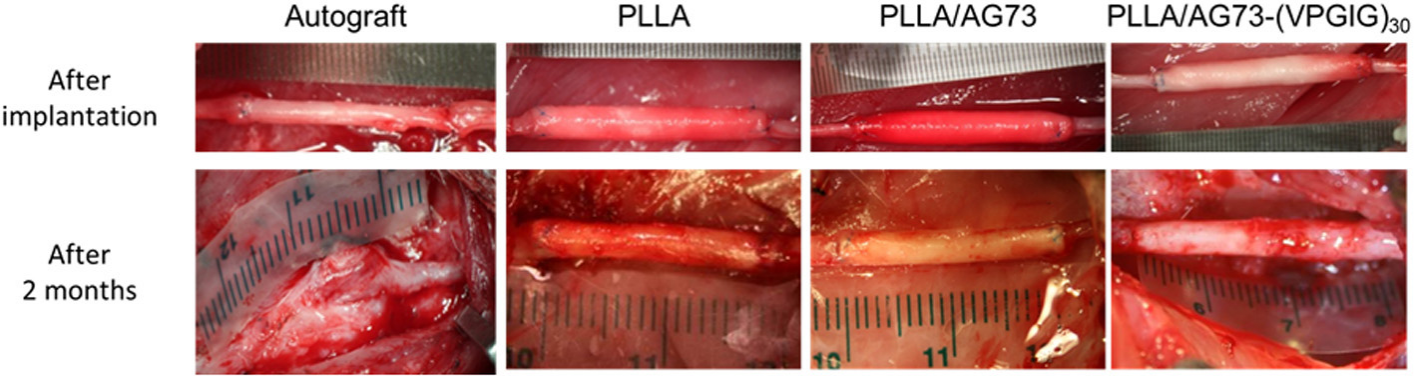
**Intraoperative photographs of autograft and micro-fibrous conduits (A) immediately after implantation and (B) 2 months after implantation**. The proximal side is shown on the left.

The electrospun microfibrous conduits had a three-layered structure, including a PLLA/PEG outer layer, a PLLA middle layer, and a PLLA, PLLA/AG73, or PLLA/ AG73-(VPGIG)_30_ inner layer, as shown in Figure 4. PEG was used in the outer layer to prevent the adhesion of surrounding tissues, because the adhesion of surrounding tissues to nerves causes painful traction neuropathies (Tashiro et al., 1989). The microfibrous conduits were approximately 500-μm thick, and the diameter of the PLLA/PEG microfiber outer layer was approximately 2.0 μm. Intraoperative photographs of the autografts and the microfibrous conduits in the rabbit tibial nerve gap were taken immediately after the implantation, and are shown in Figure 5. The conduits possessed sufficient strength for suturing and maintained their tubular shape after implantation. Two months after implantation, the nerve autografts were strongly adhered to the surrounding tissue and muscle, as shown in Figure 5. In contrast, the microfibrous conduits were easily located because the PEG in the PLLA microfibers of the outer layer had suppressed tissue adhesion. All conduits maintained their tubular structure during the 2 months, due to the slow degradation of the high molecular weight PLLA.

**Figure 6:**
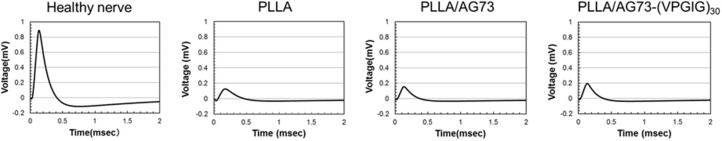
**Active potential of healthy nerve and PLLA micro-fibrous conduits 2 months after implantation**.

Reinnervation by nerve autografts or conduit implantation was analyzed by electrophysiological detection of active potentials (Figure 6). The action potential of healthy tibial nerve was 0.9 mV at 0.12 ms. The intensity and peak time of the action potential are summarized in Table 1. No action potential was detected in implanted nerve autografts, due to the abnormal adhesion of surrounding tissues, but action potentials were reproducibly detected for PLLA microfibrous conduits. In unmodified PLLA conduit, the time and intensity of the action potential were 0.19 ms and 0.09 mV, respectively. Mixing the AG73 peptide in the inner layers lightly improved the action potential, to 0.16 mV at 0.16 ms. The microfibrous conduit with a PLLA/AG73-(VPGIG)_30_ inner layer carried an action potential of 0.15 ms at 0.21 mV. This recovery is far from functional reinnervation, but nerve regeneration was slightly enhanced by mixing PLLA with AG73-(VPGIG)_30_, compared to PLLA and PLLA/AG73 nerve conduits due to the stable interaction of AG73-(VPGIG)_30_ with PLLA microfibers.

**Table 1:** **Peak time and intensity of active potentials determined using electrophysiological analysis**.

**Experimental group**	**Peak of active potential**	***N***			**Average**	***SD***
		**1**	**2**	**3**		
PLLA	Time (ms)	0.21	0.19	0.17	0.19	0.02
	Intensity (mV)	0.11	0.03	0.12	0.09	0.05
PLUVAG73	Time (ms)	0.16	0.17	0.16	0.16	0.01
	Intensity (mV)	0.28	0.13	0.08	0.16	0.10
PLUVAG73-(VPGIG)_30_	Time (ms)	0.17	0.15	0.14	0.15	0.02
	Intensity (mV)	0.24	0.19	0.19	0.21	0.03

## Conclusion

PLLA microfibers containing the AG73 peptide or the elastin-laminin mimetic protein AG73-(VPGIG)_30_ were fabricated using an electrospinning procedure. AG73-(VPGIG)_30_ was homogeneously mixed in PLLA microfibers, and the fibers were insoluble in a physiological environment. Neurite outgrowth of PC12 cells was promoted on the PLLA/AG73-(VPGIG)_30_ non-woven microfibers compared to that on the PLLA and PLLA/AG73 non-woven microfibers. In addition, we prepared electrospun microfibrous conduits with a three-layered structure: a PLLA/PEG outer layer, a PLLA middle layer, and a PLLA, PLLA/AG73, or PLLA/AG73-(VPGIG)_30_ inner layer. When microfibrous conduits were implanted into a 2.0-cm gap in an injured rabbit tibial nerve, their tubular structure was maintained after suturing. Although nerve autografts strongly adhered to the surrounding tissue, PLLA microfibrous conduits did not, owing to PEG mixed in the outer layer. The active potential was slightly improved in PLLA/AG73-(VPGIG)_30_ microfibrous conduit compared to PLLA and PLLA/AG73 microfibrous conduits. However, this recovery was insufficient to achieve functional reinnervation. Microfiber orientation has been reported to influence the differentiation and proliferation of neural stem cell (Bashur et al., 2006; Ghasemi-Mobarakeh et al., 2008). In this study, the structure of the AG73-(VPGIG)_30_ microfibers of the inner layer was random. Alignment of the inner-layer fibers of conduits is expected to enhance reinnervation. The results of this study demonstrate that the electrospun PLLA microfiber conduit with a PEG-mixed outer layer and an AG73-(VPGIG)_30_-mixed inner layershows excellent potential for enhancing nerve regeneration.

### Conflict of interest statement

The authors declare that the research was conducted in the absence of any commercial or financial relationships that could be construed as a potential conflict of interest.

## References

[B1] Ao Q. Wang A. Cao W. Zhang L. Kong L. He Q. (2006). Manufacture of multimicrotubule chitosan nerve conduits with novel molds and characterization *in vitro*. J. Biomed. Mater. Res. A 77A, 11–18 10.1002/jbm.a.30593 16345091

[B2] Archibald S. J. Shefner J. Krarup C. Madison R. D. (1995). Monkey median nerve repaired by nerve graft or collagen nerve guide tube. J. Neurosci. 15, 4109–4123 7751969 10.1523/JNEUROSCI.15-05-04109.1995 PMC6578227

[B3] Bashur C. A. Dahlgren L. A. Goldstein A. S. (2006). Effect of fiber diameter and orientation on fibroblast morphology and proliferation on electrospun poly(d,l-lactic-co-glycolic acid) meshes. Biomaterials 27, 5681–5688 10.1016/j.biomaterials.2006.07.005 16914196

[B4] Chernousov M. A. Yu W. Chen Z. Carey D. J. Strickland S. (2008). Regulation of schwann cell function by the extracellular matrix. GLIA 56, 1498–1507 10.1002/glia.20740 18803319

[B5] Freier T. Montenegro R. Koh H. S. Shoichet M. S. (2005). Chitin-based tubes for tissue engineering in the nervous system. Biomaterials 26, 4624–4632 10.1016/j.biomaterials.2004.11.040 15722132

[B6] Ghasemi-Mobarakeh L. Prabhakaran M. P. Morshed M. Nasr-Esfahani M.-H. Ramakrishna S. (2008). Electrospun poly(ε-caprolactone)/gelatin nanofibrous scaffolds for nerve tissue engineering. Biomaterials 29, 4532–4539 10.1016/j.biomaterials.2008.08.007 18757094

[B7] Hersel U. Dahmen C. Kessler H. (2003). RGD modified polymers: biomaterials for stimulated cell adhesion and beyond. Biomaterials 24, 4385–4415 10.1016/S0142-9612(03)00343-0 12922151

[B8] Huang Z. Zhang Y. Z. Kotaki M. Ramakrishna S. (2003). A review on polymer nanofibers by electrospinning and their applications in nanocomposites. Compos. Sci. Technol. 63, 2223–2253 10.1016/S0266-3538(03)00178-7

[B9] Ichihara S. Inada Y. Nakamura T. (2008). Artificial nerve tubes and their application for repair of peripheral nerve injury: an update of current concepts, ainjury. Injury 39 Suppl. 4, S29–S39 10.1016/j.injury.2008.08.029 18804584

[B10] Iwamoto Y. Robey F. A. Graf J. Sasaki M. Kleinman H. K. Yamada Y. (1987). YIGSR, a synthetic laminin pentapeptide, inhibits experimental metastasis formation. Science 238, 1132–1134 10.1126/science.2961059 2961059

[B11] Kakinoki S. Uchida S. Ehashi T. Murakami A. Yamaoka T. (2011). Surface modification of poly(l-lactic acid) nanofiber with oligo(D-lactic acid) bioactive-peptide conjugates for peripheral nerve regeneration. Polymers 3, 820–832 10.3390/polym3020820

[B12] Kakinoki S. Yamaoka T. (2010). Stable modification of poly(lactic acid) surface with neurite outgrowth-promoting peptides via hydrophobic collagen-like sequence. Acta Biomater. 6, 1925–1930 10.1016/j.actbio.2009.12.001 19969110

[B13] Kakinoki S. Yamaoka T. (2014). Thermoresponsive elastin/laminin mimicking artificial protein for modifying PLLA scaffolds in nerve regeneration. J. Mat. Chem. B. 10.1039/c4tb00305e 32261839

[B14] Lohmeyer J. A. Siemers F. Machens H.-G. Mailänder P. (2009). The clinical use of artificial nerve conduits for digital nerve repair: a prospective cohort study and literature review. J. Reconstr. Microsurg. 25, 55–61 10.1055/s-0028-1103505 19037847

[B15] Lundborg G. Dahlin L. B. Danielsen N. Gelberman R. H. Longo F. M. Powell H. C. (1982). Nerve regeneration in silicone chambers: influence of gap length and of distal stump components. Exp. Neurol. 76, 361–375 10.1016/0014-4886(82)90215-1 7095058

[B16] Lynn S. K. Yannas I. V. Bonfield W. (2004). Antigenicity and immunogenicity of collagen. J. Biomed. Mater. Res. 71B, 343–354 10.1002/jbm.b.30096 15386396

[B17] Mainil-Varlet P. Gogolewski S. Nieuwenhuis P. (1996). Long-term soft tissue reaction to various polylactides and their *in vivo* degradation. J. Mater. Sci. Mater. Med. 7, 713–721 10.1007/BF0012140614613255

[B18] Nakamura T. Inada Y. Fukuda S. Yoshitani M. Nakada A. Itoi S. (2004). Experimental study on the regeneration of peripheral nerve gaps through a polyglycolic acid-collagen (PGA-collagen) tube. Brain Res. 1027, 18–29 10.1016/j.brainres.2004.08.040 15494153

[B19] Nomizu M. Song S. Y. Kuratomi Y. Tanaka M. Kim W. H. Kleinman H. K. (1996). Active peptides from the carboxyl-terminal globular domain of laminin alpha2 and Drosophila alpha chains. FEBS Lett. 396, 37–42 10.1016/0014-5793(96)01060-5 8906862

[B20] Rao S. S. Winter J. O. (2009). Adhesion molecule-modified biomaterials for neural tissue engineering. Front. Neuroeng. 2:6 10.3389/neuro.16.006.2009 19668707 PMC2723915

[B21] Rutka J. T. Apodaca G. Stern R. Rosenblum M. (1988). The extracellular matrix of the central and peripheral nervous systems: structure and function. J. Neurosurg. 69, 155–170 10.3171/jns.1988.69.2.0155 3292716

[B22] Sieminow M. Brzezicki G. (2009). Current techniques and concepts in peripheral nerve repair. Int. Rev. Neurobiol. 87, 141–172 10.1016/S0074-7742(09)87008-6 19682637

[B23] Smit X. van Neck J. W. Afoke A. Hovius S. E. R. (2004). Reduction of neural adhesions by biodegradable autocrosslinked hyaluronic acid gel after injury of peripheral nerves: an experimental study. J. Neurosurg. 101, 648–652 10.3171/jns.2004.101.4.0648 15481720

[B24] Suzuki M. Itoh S. Yamaguchi I. Takakuda K. Kobayashi H. Shinomiya K. (2003a). Tendon chitosan tubes covalently coupled with synthesized laminin peptides facilitate nerve regeneration *in vivo*. J. Neurosci. Res. 72, 646–659 10.1002/jnr.10589 12749030

[B25] Suzuki N. Ichikawa N. Kasai S. Yamada M. Nishi N. Morioka H. (2003b). Syndecan binding sites in the laminin α1 chain G domain. Biochemistry 42, 12625–12633 10.1021/bi030014s 14580209

[B26] Tashiro K. Sephel G. C. Weeks B. Sasaki M. Martin G. R. Kleinman H. K. (1989). A synthetic peptide containing the IKVAV sequence from the a chain of laminin mediates cell attachment, migration, and neurite outgrowth. J. Biol. Chem. 264, 16174–16182 2777785

[B27] Thomas P. K. (1964). The deposition of collagen in relation to schwann cell basement membrane during peripheral nerve regeneration. J. Cell Biol. 23, 375–382 10.1083/jcb.23.2.375 14222821 PMC2106532

[B28] Toyota B. Carbonetto S. David S. (1990). A dual laminin/collagen receptor acts in peripheral nerve regeneration. Proc. Natl. Acad. Sci. U.S.A. 87, 1319–1322 10.1073/pnas.87.4.1319 2154740 PMC53466

[B29] Wang K.-K. Nemeth I. R. Seckel B. R. Chakalis-Haley D. P. Swann D. A. Kuo J.-W. (1998). Hyaluronic acid enhances peripheral nerve regeneration *in vivo*. Microsurgery 18, 270–275 9779641 10.1002/(sici)1098-2752(1998)18:4<270::aid-micr11>3.0.co;2-v

[B30] Yoshitani M. Fukuda S. Itoi S. Morino S. Tao H. Nakada A. (2007). Experimental repair of phrenic nerve using a polyglycolic acid and collagen tube. J. Thorac. Cardiovasc. Surg. 133, 726–732 10.1016/j.jtcvs.2006.08.089 17320572

